# Single-nucleus RNA sequencing reveals the shared mechanisms inducing cognitive impairment between COVID-19 and Alzheimer’s disease

**DOI:** 10.3389/fimmu.2022.967356

**Published:** 2022-09-23

**Authors:** Yifan Fu, Zhirong Guo, Yulin Wang, Haonan Zhang, Feifan Zhang, Zihao Xu, Xin Shen, Reiko T. Roppongi, Shaocong Mo, Wenchao Gu, Takahito Nakajima, Yoshito Tsushima

**Affiliations:** ^1^ School of Biological Science and Medical Engineering, Beihang University, Beijing, China; ^2^ College of Clinical, Chinese Academy of Medical Sciences and Peking Union Medical College, Beijing, China; ^3^ Department of Obstetrics and Gynecology, Peking University First Hospital, Beijing, China; ^4^ Department of Nephrology, Zhongshan Hospital, Fudan University, Shanghai, China; ^5^ Shanghai Medical College, Fudan University, Shanghai, China; ^6^ Department of Digestive Diseases, Huashan Hospital, Fudan University, Shanghai, China; ^7^ Gunma University Initiative for Advanced Research, Maebashi, Japan; ^8^ Department of Diagnostic and Interventional Radiology, University of Tsukuba, Ibaraki, Japan; ^9^ Department of Diagnostic Radiology and Nuclear Medicine, Gunma University Graduate School of Medicine, Maebashi, Japan

**Keywords:** single-nucleus RNA sequencing, Alzheimer’s disease, SARS-CoV-2, AD-high-risk scores, cognitive impairment, neuroinflammation

## Abstract

Alzheimer’s disease (AD)-like cognitive impairment, a kind of Neuro-COVID syndrome, is a reported complication of SARS-CoV-2 infection. However, the specific mechanisms remain largely unknown. Here, we integrated single-nucleus RNA-sequencing data to explore the potential shared genes and pathways that may lead to cognitive dysfunction in AD and COVID-19. We also constructed ingenuity AD-high-risk scores based on AD-high-risk genes from transcriptomic, proteomic, and Genome-Wide Association Studies (GWAS) data to identify disease-associated cell subtypes and potential targets in COVID-19 patients. We demonstrated that the primary disturbed cell populations were astrocytes and neurons between the above two dis-eases that exhibit cognitive impairment. We identified significant relationships between COVID-19 and AD involving synaptic dysfunction, neuronal damage, and neuroinflammation. Our findings may provide new insight for future studies to identify novel targets for preventive and therapeutic interventions in COVID-19 patients.

## Introduction

COVID-19, the disease caused by infection with the SARS-CoV-2 virus, has become a worldwide pandemic (1–5). Although the predominant clinical symptom is respiratory disease, the neurological presentation caused by COVID-19 is increasingly being recognized. Patients with COVID-19 commonly develop neurological symptoms ranging from anosmia, headache, fatigue, vomiting, memory impairment, and gait disorders to breathing difficulties and coma (6). Among a large cohort of more than 236,739 COVID-19 survivors, the estimated neurological or psychiatric diagnosis incidence in the following 6 months was 33.62%. Notably, the entire COVID-19 cohort had an estimated incidence of 0.67% for dementia (7). Moreover, patients with dementia were associated with elevated risk for COVID-19. Similar to dementia, evidence indicates that COVID-19 may have neurocognitive impairment mechanisms similar to those of some neurodegenerative diseases, especially Alzheimer’s disease (AD).

Identifying the potential mechanisms of COVID-19 and the resulting cognitive impairment are essential for early intervention and therapy. The above evidence prompted us to investigate the AD-like neurological symptoms associated with SARS-CoV-2 infection by exploring pathological processes related to those of AD. AD is a neurodegenerative condition that primarily affects elderly individuals with increasing incidence in recent years. AD primarily presents as progressive cognitive and behavioural impairments, including anterograde and retrograde amnesia (8). The pathogenic mechanisms associated with AD involve synaptic loss/damage, neuronal death, neuroinflammation, mitochondrial fragmentation, mitochondrial DNA damage, altered neurotransmitter levels, senile plaques, neurofibrillary tangles, and so on (9–13). However, whether SARS-CoV-2 infection affects cognitive function through the above comprehensive mechanisms is unknown, but the underlying mechanisms are worth exploring.

High-throughput multiomics datasets for patients with COVID-19 have been subjected to unbiased investigation of the pathophysiological process. Recently, single-nucleus RNA sequencing (snRNA-seq) has enabled large-scale transcriptomic profiling of single-cell characterization (14, 15). We believe that the shared mechanisms of AD and COVID-19 identified by snRNA-seq could provide novel insights into the process of cognitive dysfunction in response to SARS-CoV-2 infection. Here, we investigated the AD-like cognitive impairment associated with COVID-19 by integrating snRNA-seq datasets from two disease groups. Specifically, we developed AD-high-risk scores to identify disease-associated cell populations and novel targets in COVID-19 patients for further therapeutic investigation. The results of this study may shed light on the screening, early detection, and timely intervention of patients with COVID-19 to avoid cognitive impairment.

## Results

### Cohort selection and cross-dataset alignment

**Figure 1:**
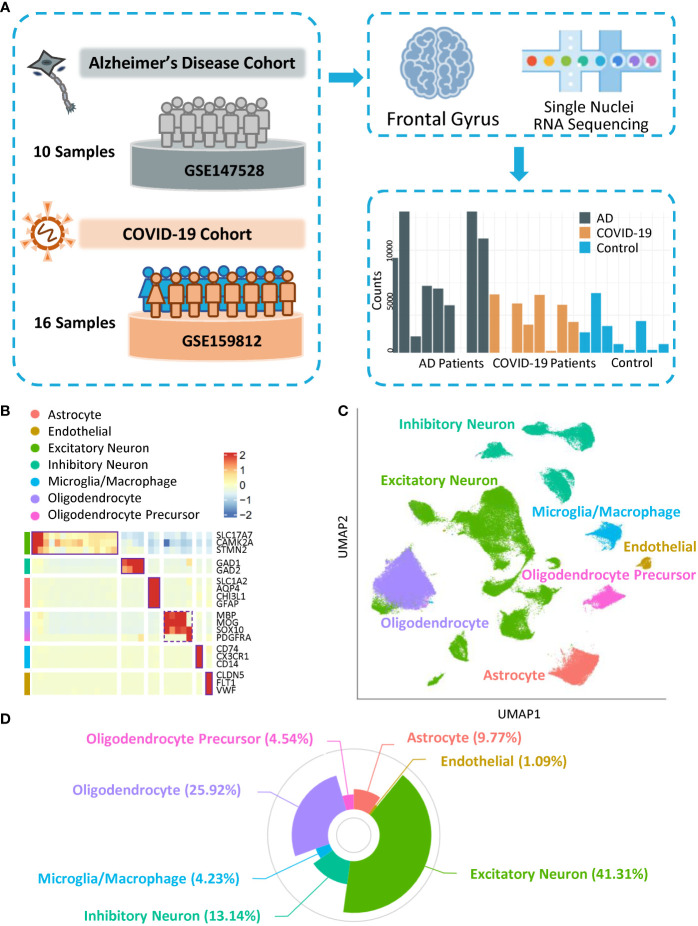
Overall landscape of cells. **(A)** Schematic of datasets from COVID-19 and Alzheimer’s Disease Cohorts. **(B)** Heatmap showing the expression of signature genes in the cells. **(C)** UMAP visualization of the cell clusters. **(D)** Composition of each subtype of cells.

To investigate cell diversity and disease-related cellular dysfunction with respect to similar pathogenic mechanisms between AD and COVID-19, we included snRNA-seq and AD high-risk genes data. In terms of brain transcriptomics data, we selected snRNA-seq data on cell nuclei extracted from the same area in two datasets (GSE147528 and GSE159812), the frontal gyrus (**Figure 1A**), from ten individuals diagnosed with AD, eight COVID-19 patients, and eight controls. Many COVID-19 patients were reported to have neurological symptoms, such as tonic-clonic symptoms, psychosyndrome, and fixed pupils (**Table S1**). Meanwhile, all APOE genotypes of AD subjects were ϵ3/ϵ3, with Braak stage (16) ranging from 0 to 6. Notably, no patients in the control group developed cognitive impairment, and no correlation between the cause of death and neuron disease was observed in either the control or COVID-19 groups. Clinical information is shown in **Table S1**. We selected AD-associated markers based on previous studies (17–21). Using cell-type-specific gene profiles, the gene list contains 368 cell-type-specific genes/proteins, including 337 in neurons and 45 from GWAS (with 14 overlapping) (**Table S2**), supporting the contribution of diverse cell types to AD pathogenesis.

### Cellular diversity of the frontal gyrus

To classify the major cell types in the frontal gyrus, we generated 143,812 single nuclei across 26 individuals. After quality control and removal of the batch effect process (**Figure S1A**), a dataset of 141,633 cells was further preclustered into 34 cell clusters (**Figure S1B**; **Table S3**) with highly consistent expression patterns across individuals (**Figure S1C**). Meanwhile, clusters with fewer than 1,000 cells were not included in downstream analysis (**Figures S1D, E**). Based on the expression pattern of marker genes of cells in the human brain, clusters with similar patterns were further aggregated into seven categories: excitatory neurons (*SLC17A7, CAMK2A*, and *STMN2*), inhibitory neurons (*GAD1* and *GAD2*), astrocytes (*SLC1A2*, *AQP4*, *CHI3L1*, and *GFAP*), oligodendrocytes (*MBP, MOG*, and *SOX10*), oligodendrocyte progenitor cells (*SOX10* and *PDGFRA*), and microglia/macrophages (*CD74, CX3CR1*, and *CD14*) and endothelial cells (*CLDN5*, *FLT1*, and *VWF*) (**Figures 1B–D**). Notably, the markers, major cell types, and cell proportions matched previous snRNA-seq data from the human cortex (22), indicating the robustness of our study. We used cell type categories to characterize the specificity of AD and COVID-19 cell cluster dysfunction and gene expression perturbations and to explore the cell type-specific potential mechanism inducing cognitive impairment.

### AD- and COVID-19-associated astrocyte subpopulations

**Figure 2:**
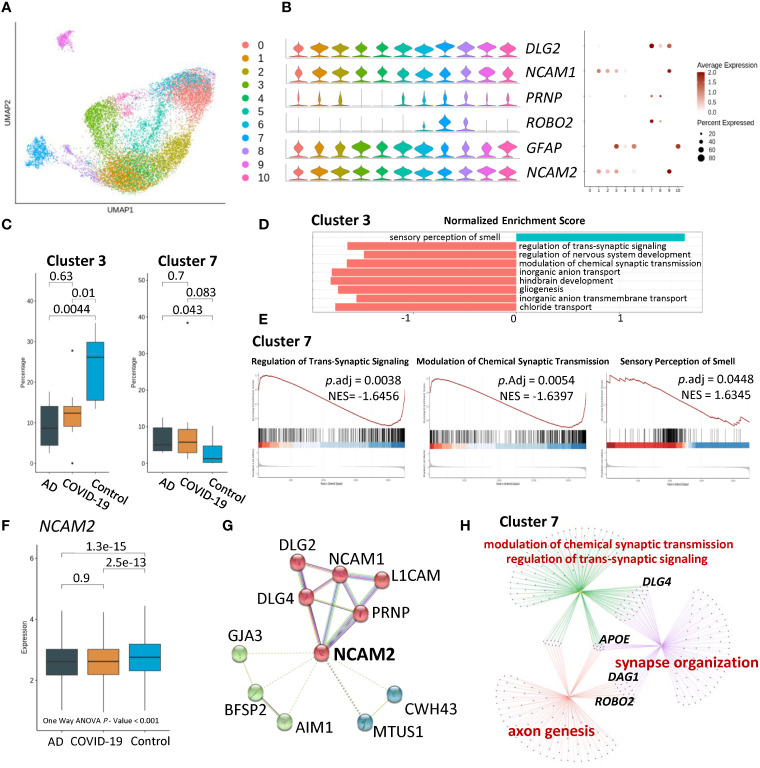
Characteristics of disease-associated astrocyte subpopulations. **(A)** UMAP embedding of all astrocytes coloured by cluster. **(B)** Violin plots and dot plots showing expression of signature genes of cells. **(C)** Composition comparison for astrocyte clusters 3 and 7, showing significant difference between groups. **(D)** GSEA result of cluster 3 showing upregulated sensory perception of the smell pathway, while downregulated several pathways related to neuron and synaptic. **(E)** GSEA result of cluster 7 showing a similar tendency in sensory of smell and synaptic-related pathways. **(F)** Boxplot of expression of NCAM2 in AD, COVID-19, CT groups, showing statistical significance. **(G)** Protein–protein interaction of NCAM2 by String Database. Proteins were clustered into 3 clusters. **(H)** Network of GO results applied in cluster 7, among which APOE, DLG4, DAG1 and ROBO2, as key proteins, are indicated.

To dissect cell-type heterogeneity, we subclustered astrocytes, resulting in ten astrocyte neuron subclusters (**Figure 2A**). Moreover, compared to the control group, AD and COVID-19 were associated with a significantly reduced percentage of cluster 3 characterized by neural cell adhesion molecule 1 (*NCAM1)*, neural cell adhesion molecule 2 (*NCAM2)*, and glial fibrillary acidic protein *(GFAP)* (**Figures 2B, C**). Therefore, we focused our analysis on cluster 3. To further understand the pathways among the disease and control groups, we performed Gene Set Enrichment Analysis (GSEA) based on Gene Ontology (GO) databases on nuclei enriched in cluster 3. GSEA demonstrated that sensory perception of the smell pathway was upregulated in cluster 3 among AD and COVID-19 patients compared with the control group. In contrast, downregulated pathways of nuclei in cluster 3 were associated with synaptic function, including regulation of transsynaptic signalling and modulation of chemical synaptic transmission (**Figures 2D, E**). As a marker of cluster 3, the average expression of *NCAM2* was significantly different among the three groups and One-way ANOVA showed the same result (**Figure 2F**). Furthermore, we focused on the *NCAM2* selected by cluster 3 and proteins that interact with *NCAM2*, including *NCAM1* and *DLG* family members (**Figure 2G**). We also observed the same significant differences in percentage in cluster 7. Dysfunctional biological processes were also downregulated in cluster 7 involving synaptic function. Comparison of cluster 7 among different groups using GO analysis revealed synapse processes involving synapse organization, modulation of chemical synaptic transmission and regulation of transsynaptic signalling (**Figure 2H**; **Table S3**).

### Cell-type-specific subclusters in neurons

**Figure 3:**
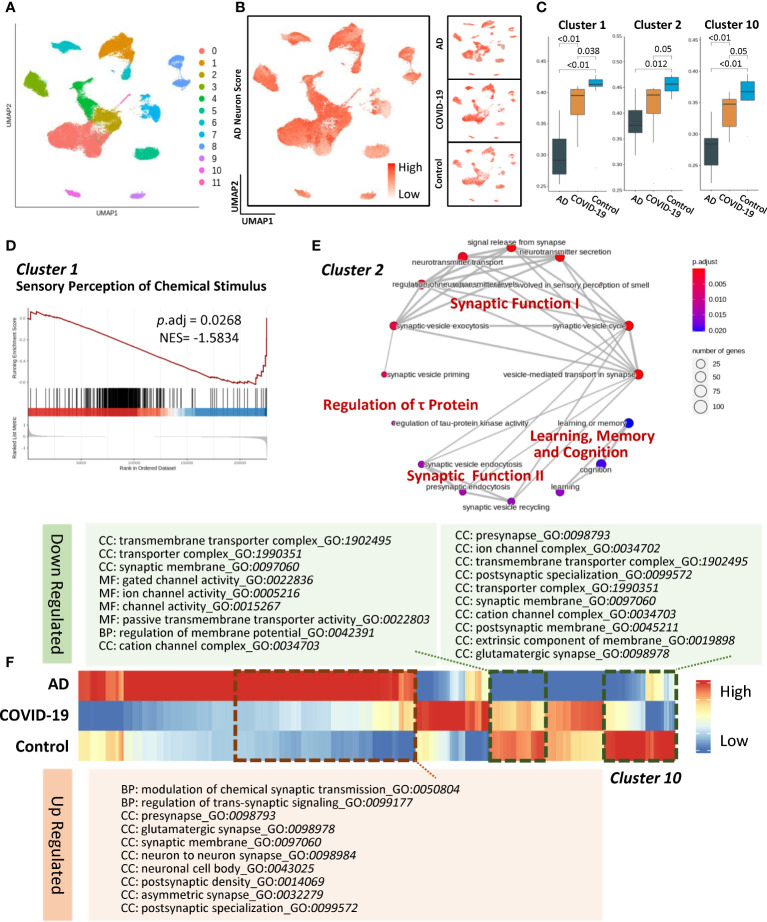
Characteristics of disease-associated excitatory neuron subpopulations. **(A)** UMAP embedding of all excitatory neurons coloured by cluster. **(B)** AD neuron score overlaid on UMAP showing the expression of AD-related gene sets of the cells. Right panels show the same value in each group. **(C)** Composition comparison for astrocyte cluster 1, cluster 2, and cluster 10 showing significant differences among groups. **(D)** GSEA result of cluster 1 showing downregulated sensory perception of chemical stimulus with adjusted P value less than 0.05. **(E)** Network of GO enrichment results in cluster 2 showing DEGs related to synaptic function, regulation of τ protein and learning, memory, and cognition pathways. **(F)** Heatmap showing expression of gene list in AD, COVID-19, CT groups and the dotted line illuminated genes with the same trend. In addition, these genes were subjected to GO enrichment and are shown as downregulated and upregulated pathways. Systematic differential analysis of gene expression between AD and COVID-19.

Given the astrocytic impairments involving the regulation of synaptic function, we next captured the neuron subtypes that are extensively affected in AD and COVID-19. In terms of excitatory neurons, the Seurat algorithm uncovered eleven subclusters (**Figure 3A**). To compare gene signatures and pathways in neuronal subpopulations, we innovatively developed an AD neuron score based on identifying cell-specific proteins and RNA in the AD brain. Briefly, a gene set investigating the expression patterns of known AD-associated genes (**Table S1**) was generated and then applied by gene set scoring analysis (as described in Method section of *Gene set scoring analysis*) to calculate the AD-high-risk scores in each subcluster (**Figure 3B**). The changes in disease-associated clusters reflected similar perturbations across the transcriptome between AD and COVID-19 (**Figure 3C**). We then hypothesized that cognitive decline might stem from changes in specific neuron clusters. In the disease group, excitatory neuron subcluster 1 represented a declining trend compared to the control group, suggesting a potential mechanism of sensory perception of chemical stimulus damage in COVID-19 (**Figure 3D**). Meanwhile, subcluster 2 specifically showed links to cognitive dysfunction (**Figure 3E**). These changes suggest that the disturbance of synaptic functional modules I and II leads to decreased signal release from synapses, neurotransmitter transport, and neurotransmitter secretion. Disease-associated subcluster 2 was also enriched in cognitive neurological symptoms, the dysfunction of which could exacerbate the degeneration of learning, memory, and cognition (**Figure 3E**). Moreover, we observed a disturbance in tau-protein regulation, consistent with the fact that tau neurofibrillary inclusions accumulate in the brain followed by neuronal loss (23). Notably, the expression profile of subcluster 10 exhibited a consistent trend among the disease and control groups (**Figure 3F**). We found that subcluster 10 was primarily involved in regulating voltage-gated channel activity, transmembrane transporter activity and synaptic functions.

### AD- and COVID-19-associated microglia and oligodendrocytes

**Figure 4:**
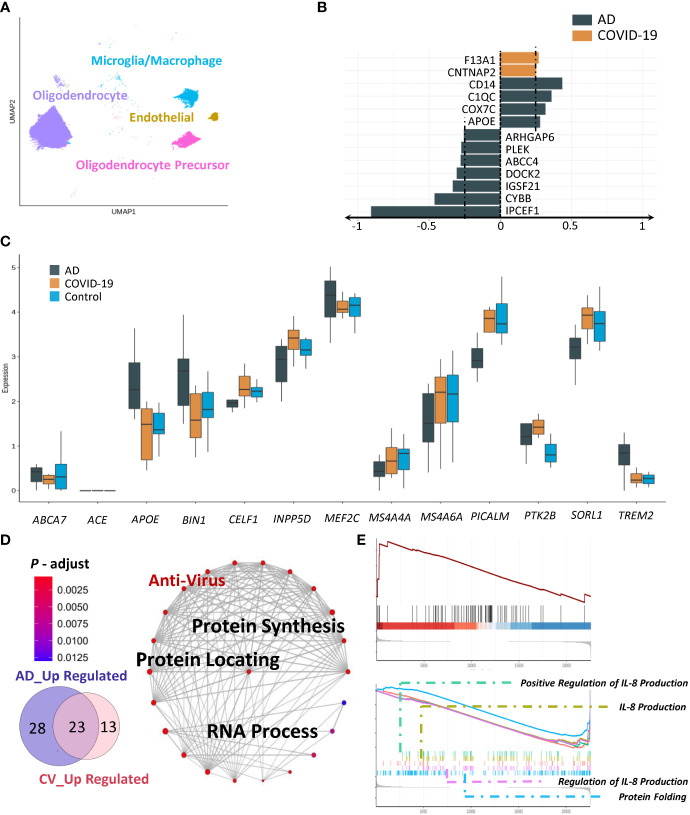
Characteristics of disease-associated subpopulations. **(A)** UMAP embedding of all other cells (Micro-glia/Macrophage, Oligodendrocyte, Endothelial, Oligodendrocyte Precursor) coloured by cell type. **(B)** Expression of AD-related genes in AD and COVID-19 groups. **(C)** Changes in disease-associated clusters. **(D, E)** GSEA and GO enrichment result of microcyte/macrophage showing both anti-virus and cognitive dysfunction.

We next sought to evaluate microglia/macrophages, oligodendrocytes, and oligodendrocyte precursors (**Figure 4A**). We first analysed microglia using our AD-high-risk scores. In the COVID-19 group, coagulation factor XIII A chain (*F13A1*) and contactin associated protein 2 (*CNTNAP2*) exhibited the strongest upregulation, with *CD14*, *C1QC*, cytochrome C oxidase subunit 7C (*COX7C*) and (*APOE*) being increased in the AD group (**Figure 4B**). Specifically, we identified three markers, apolipoprotein E (*APOE*), membrane-spanning 4-domain subfamily A member 4A (*MS4A4A*), and protein-tyrosine kinase 2-beta (*PTK2B*), which displayed the same trend in AD and COVID-19 compared to the control group according to our AD-high-risk scores (**Figure 4C**). We suspected that SARS-CoV-2 infection impacts several immune-related genes/pathways that could lead to AD-like neurologic impairment. In addition to the above genes with similar trends, there were 23 overlapping activated pathways between AD and COVID-19 (**Figure 4D**). These processes were protein synthesis, protein localization and RNA processing.

We then examined cell-type-specific gene expression involving oligodendrocytes. Although we could not detect the specific subclusters in the disease, the changes were also reflected in the pathways of AD pathogenesis-associated targets. Robust differences were identified in the association of common upregulated pathways among diseases, including chaperone-mediated protein folding and positive regulation of interleukin-8 production (**Figure 4E**). In addition, due to low abundance and fewer functional connections, we excluded endothelial cells from further analysis.

**Figure 5:**
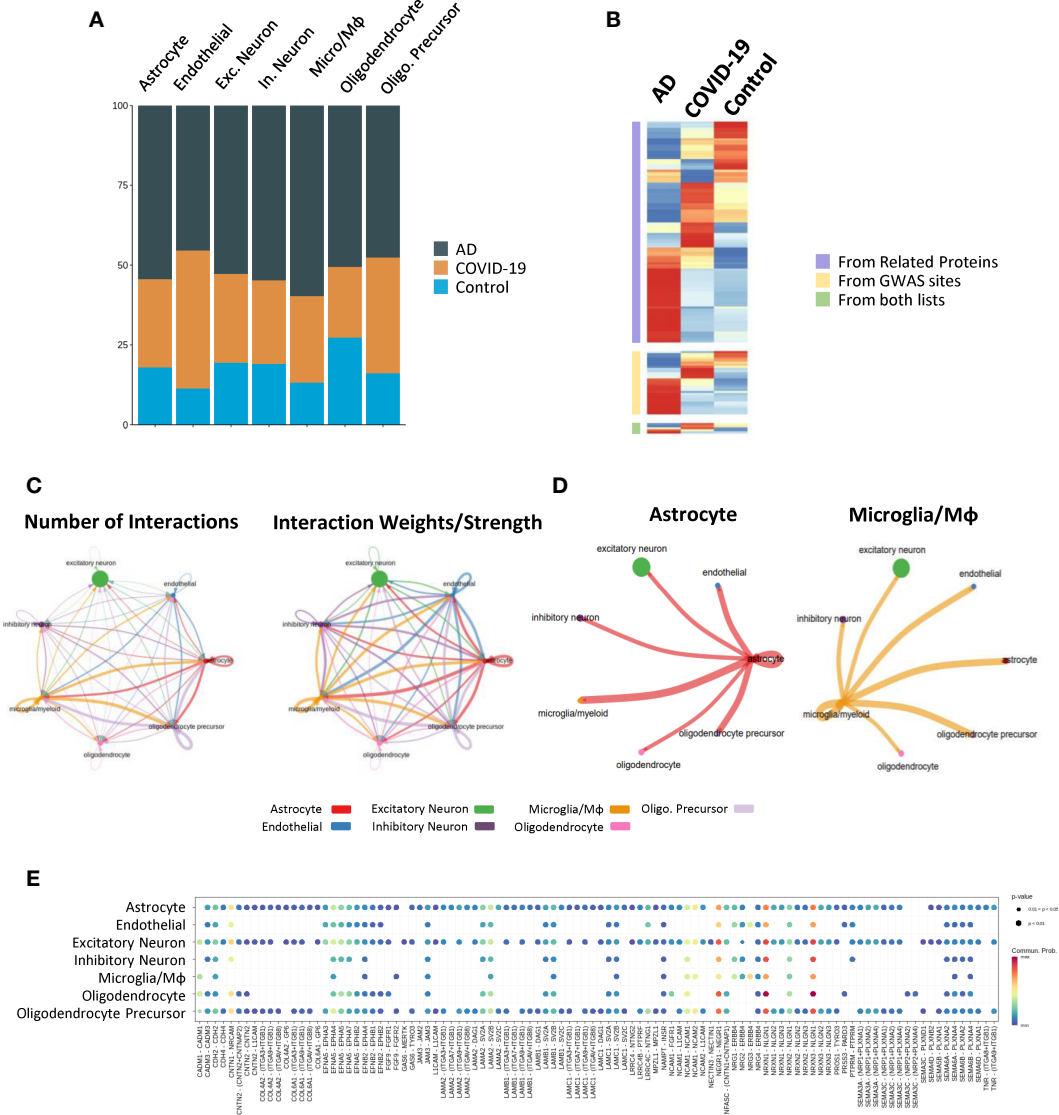
View of subtypes and cell–cell communication. **(A)** Relative abundance of each cell type in the AD, COVID-19 and control groups. **(B)** AD neuron score overlaid on UMAP showing the expression of AD-related gene sets in the cells. The right panels show the same value in each group. **(C, D)** Circle network and heatmap diagram of significant cell–cell interaction pathways. Arrows and edge colour indicate direction (ligand: receptor), and edge thickness indicates the sum of numbers (left) and weights (right) paths between populations. Network shown by cell types and heatmap by clusters. **(E)** Dot plot showing the key ligands in outgoing signalling, NCAM, the pattern of the sub-population as astrocytes.

To explore the potential pathogenesis from an overall perspective, we divided all cells into COVID-19, AD and control groups and obtained the relative abundance of each cell subtype in each group (**Figure 5A**). However, there were no significant differences between different cell types except endothelial cells. We next analysed the intersection among COVID-19 DEGs with those that have been confirmed in AD. The same trend was particularly significant, involving AD-high-risk genes from AD-specific proteins, AD-specific GWAS sites, and the above lists in the AD and COVID-19 groups (**Figure 5B**). This result suggested that COVID-19 patients may have similar changes in specific brain areas compared to AD patients, supporting our hypothesis that COVID-19 causes AD-like neuron damage in the brain.

To further clarify the underlying intercellular communications that drive cognitive impairment, we also examined the cell communication between each cell type and visualized the numbers and weights through networks using CellChat, detecting abundant ligand–receptor pairs among the 7 cell types (**Figure 5C**). As vital components of immunity, many works have demonstrated the function they carry in AD and COVID-19 (24–26). Notably, an emerging topic of interest is that Astrocyte-Microglial interactions change dramatically after inflammatory insult (27). Consistent with this point, we found that the cell communication between microglia/macrophages and astrocytes was stronger than that in other cell types in both number and strength (**Figures 5C, D**), suggesting a potential activated inflammatory reaction in COVID-19 and AD brains.

As expected, disease-specific excitatory neurons subcluster 2 exhibited strong interactions with other cells, especially astrocyte_2, 5, and 6, suggesting intercellular disturbance (**Figure 5D**). Components involved in synaptic plasticity, including *NCAM1, NCAM2, L1CAM*, and *FGFR1*, were increased during the interactions of astrocytes with other cells than the other receptor ligands, suggesting enhanced regulatory function involving synaptic plasticity (**Figure 5E**). Expression of Neurexin 1 (*NRXN1*), the function of which is to be involved in the formation of synaptic contacts, is expressed by astrocytes, and its ligands are increased in excitatory neurons as well as inhibitory neurons.

## Discussion

As COVID-19 complications, neurological symptoms have been widely reported in several recent epidemiological studies (6). Among these, AD-like cognitive impairment caused by the long-term impact of SARS-CoV-2 infection has been reported. Consistently, viral infections, such as respiratory syncytial virus and herpes simplex virus, were confirmed to be associated with AD (28). Therefore, we examined whether COVID-19 infection had potential associations with AD in an AD-like Neuro-COVID syndrome. Here, we investigated the molecular mechanism for these potential links by integrating snRNA-seq datasets. We found a strong gene-, protein- and pathway-based relationship between AD and COVID-19 involving synaptic functions, neuronal damage and neuroinflammation. Notably, innovative astrocytes are the major affected cells in AD and COVID-19. As the primary cell type in the brain, astrocytes are involved in various physiological processes, such as maintaining the blood–brain barrier (BBB) and blood flow, modulating synaptic plasticity, and regulating energy homeostasis (29). Astrocytes can detect neuronal activity and release chemical transmitters that control synaptic activity (30, 31). We simultaneously illuminated the roles of excitatory neurons, inhabiting neurons, and microglia in neuronal damage when infected with COVID-19, but endothelial cells were not investigated in this study due to our primary aim and may require further investigation.

Here, we assessed the disease-specific subpopulations in AD and COVID-19 involving transmembrane transporter activity, synaptic functions, synapse organization, and transsynaptic signalling. It is worth noting that a strong disturbance of NCAM2 indicated a potential intervention target. As an important member of the immunoglobulin superfamily of cell adhesion molecules, NCAM2 primarily participates in homophilic trans-interactions (32, 33). During synapse formation, NCAM2 participates in the molecular recognition of presynaptic and postsynaptic compartments by mechanical anchoring. Through direct or indirect interactions, it facilitates the rearrangement of the cytoskeleton, modulate calcium flow and maintains neuronal stabilization (34–36). Notably, evidence from genetic studies has confirmed the association between *NCAM2* and AD. It has been proposed that a decrease in *NCAM2* in synapses could lead to changes in cytoskeletal structures and compromise synaptic viability in AD (34, 37). Therefore, we inferred that synaptic dysfunction caused by decreased *NCAM2* may play a role in cognitive impairment among COVID-19 patients.

Neuronal damage is a major hallmark of AD. Our study also identified the disease-specific excitatory neuronal subpopulation involved in the disturbance of tau-protein regulation, consistent with the fact that tau neurofibrillary inclusions could accumulate in the brain followed by neuronal loss. Several elevated AD marker genes (e.g., *FERMT2*, *HLA-DRB1*, *GNA15*, *STAB1*, *ICA1L*, *COLGALT1*, *TNFAIP2*, *ITGAM*, *VASP*, *IDLIA*, *PVR*, *TECPR1*) were increased in the COVID-19 group. Genes that showed the same downwards trend in the AD and COVID-19 groups included *FCGR2A*, *MSR1*, *TBXAS1*, *FGL2*, *GBP2*, *PLXDC2*, *MOB3C*, *PARVG*, *SYT6*, *CFD*, *GALK1*, *NFKB2*, *CAPG*, *PLCG2*, *APOBR*, and *C1QA*. For example, as a genetic risk factor for AD, *FERMT2* controls axonal growth and synaptic plasticity in an APP-dependent manner (38). In addition, previous association studies have provided evidence for the associations of *HLA-DRB1*/*DQB1* alleles with AD (39). Meanwhile, we also observed some unique changes in COVID-19 brains such as *ICA1L*, whose protein abundance was identified as significantly associated with AD in blood and brain studies (40). A recent study based on genetic, proteomic, and transcriptomic approaches reported that the change in *ICA1L* may provide important leads to the design of future functional studies and potential drug targets for AD (41). These changes might provide new insight to exam the neuronal damage of COVID-19 in the brain.

In summary, the detected changes involving risk genes of excitatory cells based on AD-high-risk scores may provide insights into the shared pathobiology of cognitive dysfunction in COVID-19 and AD. Meanwhile, recent reports, considering the regeneration and repairability of tissue, have shown that some specific neuronal damage is not permanent and can be rehabilitated in a few months (42, 43). Moreover, some drug-based therapeutic regimens might also be helpful for treating neuronal damage (44, 45). Hence, we did not anticipate that an increasing number of patients infected by COVID-19 would suffer from AD in the long term, although more evidence is needed to confirm these findings. Regarding neuroinflammation, our AD-high-risk scores also exhibited the same trend. We focused on identifying three markers, *APOE*, *MS4A4A*, and *PTK2B*. There are several strengths in our study.

Overall, we investigated COVID-19-associated neurological cognitive manifestations using snRNA-seq. Exploring the brain heterogeneity involving cognitive impairment in COVID-19 is fundamentally challenging due to ethical obstacles and the lack of physiological *in vitro* models. Here, rare datasets from COVID-19 and AD patients using snRNA-seq technology were included. In particular, we exacted the same encephalic region, the frontal gyrus, for analysis to minimize research bias. In addition, in terms of age, sex, and status of death, the clinical characteristics of the included subjects were relatively uniform. Furthermore, we innovatively developed AD-high-risk scores based on solid AD studies involving snRNA-seq, proteomics, and GWAS sites. On the one hand, in AD areas, the above advanced method linked genotype, proteotype, and phenotype, explaining the existing comprehensive mechanism of AD. On the other hand, previous high-throughput studies in AD present a new frontier to link COVID-19-associated dementia-like symptoms and specific AD-associated cognitive impairment, accelerating the development of improved long-term prognoses in COVID-19 patients.

In summary, we identified similarities in several subtypes of frontal gyrus cells between AD and COVID-19 patients. We observed significant mechanistic overlap between AD and COVID-19 centred on synaptic functions, neuronal damage and neuroinflammation. We revealed the change in relative abundance in specific cell types marked by cell type-specific genes and illuminated the potential function and pathways they influenced by gene set enrichment. Meanwhile, the AD neuron score, AD-specific proteins, cell–cell interactions and network were also used to describe the similarity in certain cell types. The above evidence supports our hypothesis that AD and COVID-19 contribute in similar ways to neuronal dysfunction in the brain, which may help to take precautionary measures for potential neuron dysfunction and benefit the improvement of the existing therapeutic regimen for COVID-19 and Alzheimer’s disease collectively. Most importantly, the shared relationships based on genes and pathways may apply to research drug targets to prevent cognitive impairment in COVID-19. Furthermore, longitudinal and long-term clinical management as well as further mechanistic studies are warranted to control the elevated risk of SARS-CoV-2 infection in the future.

### Limitations of the study

There are several limitations of our study. First, our human AD-high-risk scores were built using high-quality and various methods, yet it is still incomplete. Second, limited by the rarity of human brain tissue, the small sample size and gender bias may affect the application of the results. Third, due to a lack of clinical information, the APOE genotypes of patients in the AD group were ϵ3/ϵ3, while those in the COVID-19 group remain unclear. Finally, the potential mechanisms of key genes and pathways may help researchers understand neurological cognitive impairment manifestations in COVID-19. However, clinical and functional studies are warranted to classify the above relationships.

### Data and code availability

The datasets and code used and/or analyzed during the current study are available from the corresponding author on reasonable request.

## Methods

### Database of snRNA-seq

To identify genetic changes in the brains of COVID-19 patients, we used two single nuclear RNA-seq datasets from the Gene Expression Omnibus (GEO) database of the National Center for Biotechnology Information (NCBI) (https://www.ncbi.nlm.nih.gov/geo/). The GEO accession is GSE147528, which is transcriptional profiling of postmortem brain tissue from donors spanning the range of Alzheimer’s disease progression, and GSE159812 includes single-nuclei RNA transcriptomes of prefrontal cortex and choroid plexus tissue obtained from postmortem brains of COVID-19, influenza, and nonviral control patients using the 10x Genomics Drop-seq gene expression kit. The datasets were published by Leng et al. (14) and Yang et al. (15), respectively. In these datasets, 10 AD subjects were included from GSE147528, and 8 subjects with COVID-19 together with 8 healthy control subjects were included from the GSE159812 dataset.

### SnRNA-seq quality control and correction of batch effect

The following criteria were applied to each cell in both datasets after being merged using the *merge* function in R. Cells with fewer than 1000 UMI counts and 500 detected genes were filtered, as well as cells with more than 10% mitochondrial gene and 15% ribosome gene counts. After quality control, a total of 141,633 cells remained. To integrate cells into a shared space from different GEO datasets for subsequent clustering, we used the Harmony algorithm to remove the batch effect (46).

### Detection and processing of snRNA-seq data

After quality control, all cells were used for dimension reduction and unsupervised clustering using the Seurat R Package. In brief, cells were normalized using the *LogNormalize* function with the *vst* method, and the mean and variance of each gene were calculated using *FindVariableFeatures*. Next, the data were scaled using the *ScaleData* function to remove the effect of genes and mitochondrial or ribosomal genes. The top 3000 genes with the highest variance were selected, and the dimensionality of the data was first reduced by principal component analysis (PCA) with 30 components, after which cells with similar expression levels were clustered using the *FindClusters* function with appropriate resolution rates (**Table S3**). The shared nearest neighbour (SNN) algorithm was utilized, and then we constructed a graph with k nearest neighbours, optimized it using the Louvain algorithm, and finally visualized the cell clusters with uniform manifold approximation and projection (UMAP). All of the above functions were from the Seurat R Package 4.0 (47).

### Differential gene expression and cell annotations

Differential gene expression analysis was performed to identify marker genes for different cell clusters using several approaches to better determine the subtype of clusters. We applied the *FindAllMarkers* function in the Seurat R Package to identify representative markers of each cluster, which were annotated by markers from previous work, as shown in **Figure 1B** (14, 15). Using the *pheatmap* function from the ComplexHeatmap R Package (48), a heatmap was visualized for the indicated cells and marker genes for annotation.

### Protein–protein interaction (PPI)

Using the STRING database (https://string-db.org, Version 11.5), PPI networks were generated based on the proteins we detected, such as NCAM2. Then, k-means clustering was used for grouping the proteins into 3 clusters.

### Gene ontology and pathway enrichment analysis

Gene set enrichment analysis (GSEA) is a powerful tool that provides the potential to interpret biological insights such as gene symbols or Gene IDs based on prior knowledge. GSEA based on Gene Ontology, functional enrichment (biological processes, cellular components, and molecular functions) and pathway enrichment studies were performed using the clusterprofiler R package (v 4.0.5) (49) to characterize the biological mechanisms and signalling pathways of shared DEGs. At the same time, visualization of functional profiles for genes and gene clusters of GSEA results was performed using the enrichplot R package (v 1.12.3) as previously (50).The *P* value (applied Benjamini–Hochberg correction to control the rate of false discovery) cut-off criteria were set as 0.05 for each listed pathway. All results are shown in **Table S3**.

### Gene set scoring analysis

Briefly, we generated AD-high-risk gene from existing studies performed by proteomics and GWAS. First, we collected gene markers from the existing studies which combined COVID-19 and AD together (51). Besides, to further determined whether there was shared mechanism between AD and COVID-19 in protein level, we included a brain proteomics converted to gene (17). Finally, we combined all these genes to generate AD-associated markers. Then, we transformed this gene list into a gene set, and the AD-high-risk scores were evaluated using the *AddModuleScore* function from the Seurat R Package. Then, we obtained the score and cluster data and calculated the neuron score in each patient. All patients’ neuron scores were grouped by clusters, and we further applied the Wilcoxon rank sum test in each cluster to obtain the statistical parameters and p values, adjusted by Benjamini & Hochberg correction. All clusters with adjusted p values less than 0.05 will be discussed in the next analysis. The neuron score threshold was applied to all cell types equally.

### Cell–cell communication analysis

To identify and visualize the possible cell–cell interactions in terms of the neuron interaction between either the selected clusters of the very subtype or between these subtypes evaluated by several approaches in our work, we employed the CellChat R Package induced by Jin et al. (52). Briefly, based on the expression of known ligand–receptor pairs in different cell types, we inferred by CellChat. We followed the official workflow and loaded the normalized counts into CellChat and applied the preprocessing functions *identifyOverExpressedGenes*, *identifyOverExpressedInteractions*, and *projectData* using a standard parameter set. For the primary analyses, the core functions *computeCommunProb*, *computeCommunProbPathway*, and *aggregateNet* were applied using standard parameters and fixed randomization seeds. Finally, to determine the senders and receivers in the network, the function *netAnalysis_signallingRole* was applied to the netP data slot.

### Statistics

To determine the typical gene marker of each cluster, we used the *FindMarkers* function from the Seurat R package with default parameters. The average log2-fold change (average log2FC) was calculated based on the mean of the same cell subtype for each cluster. Two-sided unpaired Welch’s t test was performed for each pair of compared groups, and adjusted p values were calculated using Benjamini & Hochberg correction. Significantly altered pathways were selected using the criteria of an adjusted p value less than 0.05 and an absolute average log2FC larger than 0.25. Meanwhile, the graphs were plotted using ggplot2 R Package, ggsci R Package, ggpubr R Package and cowplot R Package.

## Data availability statement

The datasets presented in this study can be found in online repositories. The names of the repository/repositories and accession number(s) can be found below: Gene Expression Omnibus, GSE147528, GSE159812.

## Author contributions

Conceptualization, YF, ZG, SM, and WG; data curation, YF, ZG, and YW; methodology, WG; supervision, WG, SM, TN and YT; visualization, YF, FZ, ZG, and YW; writing – original draft, YF, ZG, and YW; writing – review and editing, ZX, XS, RR, and SM. All authors contributed to the article and approved the submitted version.

## Acknowledgments

We thank all other researchers who participated in this study. We appreciate all our team members at Bioinfo_composer, the leading bioinformatics platform in China, for their selfless help. We also appreciate the support of the High-performance Computing Platform of Peking University. We wish to thank Prof. Chen Sang from Beihang University, for her valuable advice on experimental design.

## Conflict of interest

The authors declare that the research was conducted in the absence of any commercial or financial relationships that could be construed as a potential conflict of interest.

## Publisher’s note

All claims expressed in this article are solely those of the authors and do not necessarily represent those of their affiliated organizations, or those of the publisher, the editors and the reviewers. Any product that may be evaluated in this article, or claim that may be made by its manufacturer, is not guaranteed or endorsed by the publisher.

## Supplementary material

Supplementary Table 1 Clinical Information.

Supplementary Table 2 Alzheimer’s Disease biomarkers.

Supplementary Table 3 Gene Oncology list.

Supplementary Table 4 Materials and Methods.

Supplementary Figure 1 Pre-Proceed of datasets. **(A)** Datasets proceed by Harmony to remove batch effect. Cells from several patients located at intensive position. **(B, C)** UMAP plots showing cells in each meta clusters **(B)** and each datasets**(C–E)** Bar plot showing the contribution of COVID 19/Non COVID 19(AD/Non AD) patients to each meta cluster.

Supplementary Figure 2 Cell-cell interaction. **(A)** Cell communicate among all cells. **(B)** Cell communicate in each cell types. **(C)** Cell communicate in the cell subclusters we focused on(astrocyte subcluster 3 and excitatory neuron subcluster.

Supplementary Figure 3 Heatmap diagram of significant cell-cell interaction pathways.
