# Supplementary figures and images for: Single-nucleus RNA sequencing reveals the shared mechanisms inducing cognitive impairment between COVID-19 and Alzheimer’s disease
Source: Front Immunol. 2022 Sep 23;13:967356. doi: 10.3389/fimmu.2022.967356 (PMC9538863; doi:10.3389/fimmu.2022.967356)

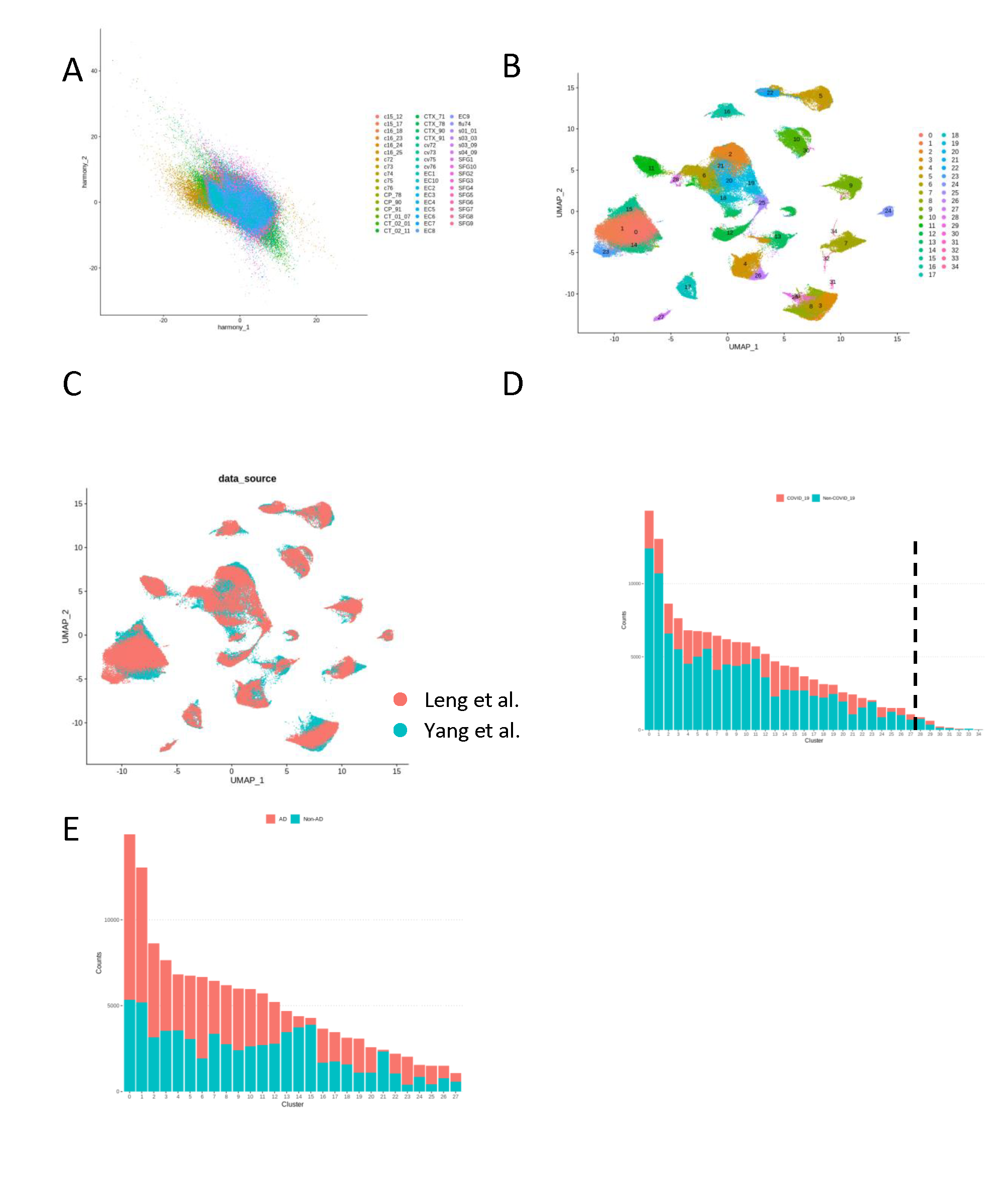

Supplement: Supplementary Figure 1 — Pre-Proceed of datasets. (A) Datasets proceed by Harmony to remove batch effect. Cells from several patients located at intensive position. (B, C) UMAP plots showing cells in each meta clusters (B) and each datasets(C–E) Bar plot showing the contribution of COVID 19/Non COVID 19(AD/Non AD) patients to each meta cluster. [file Image_1.tif]

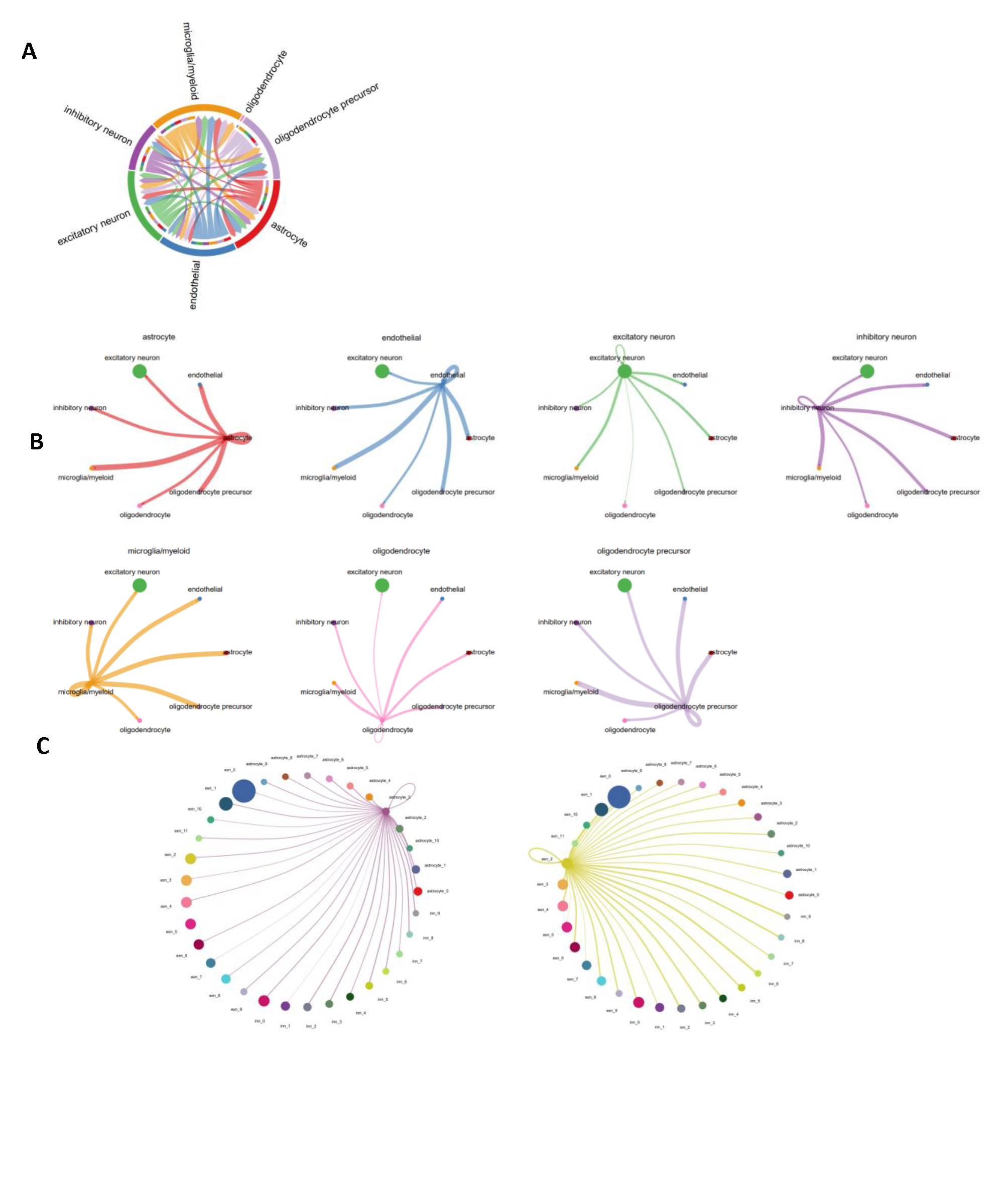

Supplement: Supplementary Figure 2 — Cell-cell interaction. (A) Cell communicate among all cells. (B) Cell communicate in each cell types. (C) Cell communicate in the cell subclusters we focused on(astrocyte subcluster 3 and excitatory neuron subcluster. [file Image_2.tif]

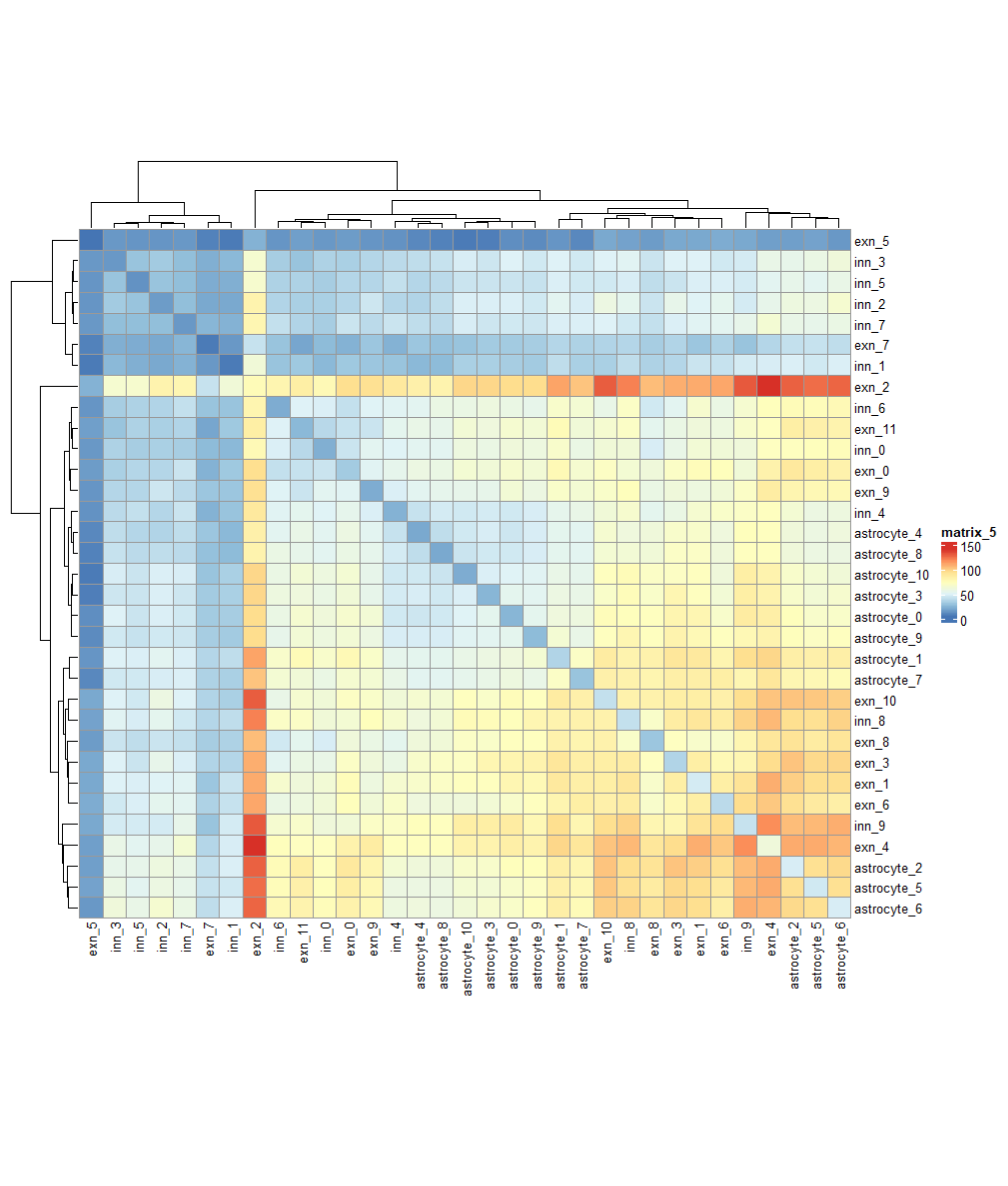

Supplement: Supplementary Figure 3 — Heatmap diagram of significant cell-cell interaction pathways. [file Image_3.tif]
